# Supplementary material for: COVID-19 Associated Contact Restrictions in Germany: Marked Decline in Children’s Outpatient Visits for Infectious Diseases without Increasing Visits for Mental Health Disorders
Source: Children (Basel). 2021 Aug 25;8(9):728. doi: 10.3390/children8090728 (PMC8471230; doi:10.3390/children8090728)
Supplement: Supplementary file 1 [file children-08-00728-s001.zip › children-1323329-SI.pdf]

children

Article

COVID-19 Associated Contact Restrictions in Germany: Marked Decline in Children's Outpatient Visits for Infectious Diseases without Increasing Visits for Mental Health Disorders

Mara Barschkett[1], Berthold Koletzko[2], C. Katharina Spiess[1]

[1] Department of Education and Family, German Institute for Economic Research (DIW), Berlin and Freie Universität Berlin, Germany; mbarschkett@diw.de; kspiess@diw.de
[2] LMU - Ludwig-Maximilians-Universität Munich, Dept. Pediatrics, Dr. von Hauner Children's Hospital, LMU University Hospital, Munich, Germany; berthold.koletzko@med.uni-muenchen.de
* Correspondence: berthold.koletzko@med.uni-muenchen.de; Prof. Berthold Koletzko, Else Kröner Senior Professor of Pediatrics, LMU - Ludwig-Maximilians-Universität Munich, Dept. Pediatrics, Dr. von Hauner Children's Hospital, LMU University Hospital, Lindwurmstr. 4, 80337 Munich, Germany, Tel: +49 89 44005 2826

Citation: Barschkett, M.; Koletzko, B.; Spiess, C.K. COVID-19 Associated Contact Restrictions in Germany: Marked Decline in Children's Outpatient Visits for Infectious Diseases without Increasing Visits for Mental Health Disorders. *Children* **2021**, *8*, 728. https://doi.org/10.3390/children8090728

Academic Editor: Benedetto Vitiello

Received: 16 July 2021
Accepted: 20 August 2021
Published: 24 August 2021

Publisher's Note: MDPI stays neutral with regard to jurisdictional claims in published maps and institutional affiliations.

Copyright: © 2021 by the authors. Licensee MDPI, Basel, Switzerland. This article is an open access article distributed under the terms and conditions of the Creative Commons Attribution (CC BY) license (http://creativecommons.org/licenses/by/4.0/).

Supplementary Material

Table S1. Number of outpatient doctor's visits per child, and frequency of selected diagnoses (ICD 10 codes) during Q2 2019 (control) and Q2 2020 (pandemic) for all 1-12 year old children in Germany with statutory health insurance who presented to a physician's office between January 2019 and June 2020. CI = Confidence Intervals, , OLS = binary Ordinary Least Square model.

Age	1-12 years		
Year	2019	2020	P<br>CI
	Raw means		OLS
Children	8,290,904	8,499,507	
**Outpatient visits per child**	0.7147 ± 0.4970	0.5888 ± 0.5240	< 0.0001<br>(-0.13,-0.13)
**Infections**			
Infectious and parasitic diseases	0.1593 ± 0.3360	0.0784 ± 0.2688	< 0.0001<br>(-0.08,-0.08)
Infectious intestinal diseases	0.0341 ± 0.1814	0.0091 ± 0.0948	< 0.0001<br>(-0.03,-0,02)
Diseases of the middle ear and mastoid	0.0605 ± 0.2383	0.0178 ± 0.1321	< 0.0001<br>(-0.04,-0.04)
Diseases of the respiratory system	0.2827 ± 0.4503	0.1380 ± 0.3449	< 0.0001<br>(-0.15, -0.14)
Acute upper respiratory tract infections	0.1750 ± 0.3800	0.0603 ± 0.2381	< 0.0001<br>(-0.12,-0.11)
Streptococcus Angina	0.0292 ± 0.1683	0.0065 ± 0.0806	< 0.0001

			(-0.02,-0.02)
**Injuries**			
Injuries	0.0436 ± 0.2042	0.0362 ± 0.1868	< 0.0001 (-0.01,-0.01)
**Chronic physical diseases**			
Diabetes	0.0012 ± 0.0350	0.0011 ± 0.0337	< 0.0001 (-0.00,- 0.00)
Celiac disease	0.0014 ± 0.0380	0.0012 ± 0.0350	< 0.0001 (-0.00, -0.00)
Hay fever	0.0321 ± 0.1763	0.0304 ± 0.1716	< 0.0001 (-0.00,-0.00)
**Mental and Behavioral Disorders**			
All mental and behavioral disorders	0.1585 ± 0.3652	0.1406 ± 0.3476	< 0.0001 (-0.02,-0.02)
Neurotic, stress and somatoform disorders	0.0185 ± 0.1349	0.0160 ± 0.1257	< 0.0001 (-0.00, -0.00)
Personality and behavioral disorders	0.0050 ± 0.0706	0.0041 ± 0.0639	n.s. (-0.00,-0.00)
Developmental Disabilities	0.1122 ± 0.3156	0.1001 ± 0.3001	< 0.0001 (-0.01,-0.01)
Behavioral & emotional disorders with onset in childhood & adolescence.	0.0595 ± 0.2365	0.0527 ± 0.2235	< 0.0001 (-0.01, -0.01)
